# Memoir of the Life and Medical Opinions of John Armstrong, M.D. Formerly Physician to the Fever Institution of London, &c. To Which Is Added, an Inquiry into the Facts Connected with Those Forms of Fever Attributed to Malaria or Marsh Effluvium

**Published:** 1836-01

**Authors:** 


### Art. II.

*Memoir of the Life and Medical Opinions of* John Armstrong, m.d., *formerly Physician to the Fever Institution of London; author of "Practical Illustrations of Typhus and Scarlet Fever," &c. &c. To which is added, an Inquiry into the Facts connected with those Forms of Fever attributed to Malaria or Marsh Effluvium.* By Francis Boott,, m.d. &c. &c. —2 vols. 8vo. pp. 616, 726. *London*, 1833-34. Baldwin; and Rainford.

*Lectures on the Morbid Anatomy, Nature, and Treatment of Acute and Chronic Diseases, delivered in the Theatre of Anatomy, Webb street, by the late* John Armstrong, m.d., &c. &c. Edited by Joseph Rix, Member of the Royal College of Surgeons, London.—8vo. pp. 851. *London*, 1834.

Although one of these works has been two years before the public, we cannot resist the temptation of arresting, for a brief space, the attention of the reader, eager perhaps in the pursuit of novelty and ambition, and of directing it to the Life and Works of one for whom novelty and ambition had considerable charms, and whose life and death afford not a few lessons to all who are following in the same path.

There has seldom existed a physician concerning whose real character and objects opinions varied more widely than concerning those of Dr. Armstrong. His zealous friends spoke, and yet speak, of him as his accomplished biographer does, as "one whose labours were directed to the promotion of the common good of mankind, and whose sagacity has thrown light on the nature and treatment of human maladies." They admired his genius, his elevated views, his simplicity of mind, and the virtues of his heart. Yet, strange to say, there were not a few persons, who had been intimate with Dr. Armstrong after his removal to London, and who looked upon him as a man of moderate ability, of great self-confidence, dominated by a restless and ill-regulated ambition, and in his friendships capricious, if not insincere. The same diversity of opinion existed respecting his qualifications as a practitioner. His admirers regarded him as a model of perspicuity and judgment; whilst not a few of his professional brethren looked upon him as equally visionary and unstable. Even at this day, the sentences in which we have embodied these conflicting views, will be read with indignation or with pleasure, by many who will deem the favorable view partial or absurd, or the unfavorable view unjust or false. Difficult as it is to arrive at a just estimate of the merits of one whose merits have been so much disputed, we may now perhaps, when death has removed him alike from his friends and his enemies, be enabled to come at some correct conclusion. His history, short as it is, and his death, which occurred in the prime of his life, and in the midst of all that is generally considered to make life desirable, may at least be contemplated with some advantage to the living, yet engaged in schemes which animated him, and sustained by hopes which only left him with his latest breath.

The origin and early education of men, however lost sight of amidst advantages subsequently enjoyed and honours attained, exercise, we believe, on all a continual influence: difficult to be traced, it may be, in the public deportment of individuals, but felt and known by every man who reflects upon himself; and, if not ever active, at least often operating on the conduct and feelings. The parents of Armstrong were persons of humble origin, but of integrity and respectability in the best sense. He passed a few years in a country school of indifferent pretensions; and at eight years of age, having not yet acquired the commonest rudiments of knowledge, was placed under the care of a clergyman of the United Secession Church of Scotland. Here he seems to have evinced talents, and to have made great progress; and it speaks well, both for the master and the pupil, that, after passing eight years at the school, the latter still delighted to avail himself of his old preceptor's lessons, during those parts of the year in which he was not engaged in learning his profession in Edinburgh.

On finally leaving school, having shewn a predilection for the medical profession, he was placed for a short time with a country surgeon; but, either disgusted with the disadvantages at that time inseparable from such arrangements, or for some other reason, and against the wishes of his parents, he left that situation, and led a desultory and almost an idle life from the age of sixteen to nineteen; years which, in the life of a youth of talent and imagination, leave little record save in the memory of the individual; years in which the mind begins to exert its powers, and a love of mental pleasures exists, more pure and delicious than all that later years have to bestow. The most useful part of any man's biography is exactly that relating to such years; but it is rarely to be come at, for no second person can indite it. The course of reading pursued, the daily companions and their characters, the customary division of time, the conversations, the designs cherished, the essays commenced, the blissful visions, occupying long days of holiday solitude; these are the general foundations of the future man, whom the world's business is soon to employ, and the world's difficulties are soon to perplex.

In this dreamy and unemployed period of his life, young Armstrong formed many visionary schemes; and his nascent ambition displayed itself in the desire which has deluded so many, and made so many wretched for life, of "going to London to seek employment in some literary occupation." This desire is peculiarly incidental to those young men who have enjoyed but few early advantages of education, but who possess talents and susceptibility. They feel their own power, and tracing it to no particular care of culture, they doubtless somewhat over-estimate it. We think this tendency to extreme self-estimation, conjoined with a want of acquaintance with other men's proficiency, is visible in all the future progress of Dr. Armstrong. If, however, it now and then misleads, its entire

D 2

absence, which a public education often effects, is much more prejudicial to the future efforts of the individual: a cold and hopeless indolence besets the mind; a bigoted admiration of certain men and certain opinions, and a timidity which forbids every independent exertion. To us, therefore, there is no part of Dr. Armstrong's early life more interesting than that in which he wandered about the neighbourhood of Sunderland; wrote fugitive pieces of poetry; and meditated a tragedy on the story of Boethius; a design to which he sometimes reverted, when immersed in the bustle and business of London, with a wish for that leisure which was incompatible with other wishes that had then risen up within his breast.

When a few years had been passed in this way, not with regular industry, but yet not fruitlessly, young Armstrong was enabled, chiefly by the economy and management of a most excellent mother, to go to Edinburgh, and study for a degree in medicine; and his gratitude to this admirable parent seems to have been fervent and durable. The number of young men sent to their studies in this manner in Edinburgh, and even in London, is, we apprehend, considerable; and we can never look upon the youthful, ardent, and hopeful countenances of the junior students at the commencement of each session, without our thoughts being carried to their several homes, where efforts are making, and sacrifices, of which young students are not always mindful,—and which give something of solemnity to the tacit engagements so boldly entered upon by their public teachers, in years which are to determine whether or not those sacrifices are to be made, and not a few parental anxieties to be endured, in vain. Like many a studious youth, Armstrong was compelled, by his narrow resources, rigidly to confine his studies to those prescribed as preliminary to taking a degree; and, whatever of temporary regret this might occasion him, he was perhaps indebted for his future professional celebrity to this necessity of restraining an attention which might otherwise have been too discursively employed. Medicine is a jealous science, and will only be courted for its own sake; rewarding with niggardly hand those followers who turn aside too often to the attractive fields of literature, or even to other and severer, or more exact and satisfactory, paths of enquiry.

At the time when Armstrong was an Edinburgh student, the chair of Medicine was filled by Dr. Gregory, whose masculine understanding, great eloquence, and the judgment with which he directed the attention of the student to the principles of his profession, were combined with what some have considered too great a disregard for the supposed novelties which were published in his later years. We confess ourselves to be inadequate judges of his merits; for we can never mention his name without the warmest feelings of gratitude for those never-to-be-forgotton lessons, of which we feel the value more as experience becomes more advanced. The wisdom, the manliness, the learning without pedantry, the scorn and contempt of quackery, and all the other recommendations of that great and accomplished teacher, contributed for a long period to keep up the dignity of an Edinburgh degree, and to maintain the proper rank of the physician.

There were, in our time, those students who were insensible to the elevated qualities of such a teacher, and who regarded him as a mere commentator on Cullen, and Cullen himself as little better than a dreamer. It does certainly excite our surprise to find that of all the eminent medical men with whom Edinburgh abounded in the years of Armstrong's studies there, the chief object of his admiration should have been, not Gregory, but Dr. Hamilton, the author of the book on Purgative Medicines. That Dr. Hamilton had the merit of shewng that purgative medicines might be safely and advantageously used in various forms of fever, and several other disorders in which their administration was in his time considered hurtful or dangerous, is willingly conceded. His precepts concerning the employment of this class of medicines are eminently judicious. His application to his purgative doctrine in his hospital practice was, however, indiscriminate; and attended in many cases, especially of fever, dysentery, and inflammatory diarrhœa, with such palpable fatality as none but a practitioner devoted to a single idea could possibly have overlooked. As a *teacher* of practice, in the course of his hospital duty, a regard for truth compels us to say, that he was little calculated to assist the learner: he was supercilious, never addressed a word of explanation to the students who followed him up and down stairs for months or years; and did all in his power, by low tones and a mystic deportment, to prevent their knowing what he deemed to be the actual state of the patient, or what he prescribed. Yet, to some students, his compendious plan of medicine recommended itself very strongly; its simplicity was enchanting; it required no reflection; and to this day, in every province in England, the injudicious and habitual prescription of laxatives, or the violent and obstinate employment of drastic medicaments, daily proclaims the durability of his narrow doctrine and erroneous example.

Almost every boasted improvement in practical medicine proves, on examination, to be a mere resuscitation of some old practice which has been for a time neglected; and Dr. Hamilton's application of purgatives to fever is often alluded to as if before his time no such practice had been known; whereas, Baglivi, Borelli, and Sydenham, advocated such treatment, and, long before them, Aretæus, Galen, and even Hippocrates. The use of violent purgatives seems to have brought the practice into discredit, and the termination of some fevers by profuse prespirations must have induced occasional doubts of the propriety of endeavouring to relieve entirely by the bowels. The frequent occurrence of diarrhœa, without relief, in fevers of the most intractable character, must also have deterred judicious practitioners from wholly relying on intestinal evacuants:

and we cannot but think that the recommendation, in the first edition of Dr. Armstrong's work on fever, that seldom less than four or five alvine evacuations should be daily procured during the stage of excitement, was one of the axioms he had borrowed without reflection, rather than established by observation as salutary, or even not pernicious.

Dr. Armstrong took his degree in 1807, when only twenty-three years of age, after three winters' study, and with little previous medical education. He immediately removed to Bishopwearmouth, and commenced practice as a physician; living in lodgings, and being almost wholly dependent on success in his profession. Not long afterward, the application of Dr. Hamilton's purgative system proved of singular advantage to him. An affluent resident of a country town, and probably living the usual easy and indulgent life of affluent gentlemen in the provinces, had for two years been subject to occasional attacks of diarrhœa, and was cured by a mild course of laxatives, prescribed by Dr. Armstrong. Dr. Hamilton happened to pass through Sunderland in a day or two after the young physician had been consulted about this case, and with a liberality which was, we believe, characteristic of him, declined seeing the patient and depriving his junior of the credit which he felt assured would be the result of the plan already adopted. The patient was cured, and, as he had reason to be, abundantly grateful to Dr. Armstrong, whose success at Sunderland seems to have been established in a great degree by his good offices. Dr. Armstrong remained five years in the north of England; and, when his early age is remembered, his success, which was very considerable, must be allowed to argue most favorably for his abilities, his perseverance, and his general character. In his twenty-eighth year, and when he had only been four years in practice, he was enabled to remove to a large house at Bishopwearmouth, and to keep his carriage. In the same year he married the daughter of Charles Spearman, Esq., of Thornley, near Durham. Thus at a time of life when few physicians have the comfort of being settled in practice, and fewer still any certain prospect of obtaining a competency, (a prospect which Dr. Baillie did not enjoy until after forty,) we find Dr. Armstrong in the possession of much provincial celebrity, with all the solid advantages arising out of it.

But Dr. Armstrong's mind was too active to be content with a limited sphere of duties, and his aspirations were unsatisfied with the mere fame of a country physician. He possessed little or no medical learning, but he never had been reminded of the want of it. He was a diligent observer of disease, and his observations appeared to himself to be both original and valuable. He therefore began to publish, and the reception of his writings by the public was for a time such as strongly to confirm these favorable views of himself. His papers in the ninth volume of the Edinburgh Medical and Surgical Journal, on Brain-fever from Intoxication, and on

Diseased Cervical Vertebræ, are characteristic of his powers of minute description, and of his love of detail: peculiarities which were equally observable in his conversation: they also exhibit him in the light of a careful and reflecting practitioner. His language, even in these his first compositions, is fluent and correct; but there is, as usually happens with unlettered men, a tinge of affectation observable in his various and not always very important references, including one to Paley's Natural Theology, to warrant the somewhat vague and superfluous announcement that "the great energies of nature are known to us only by their effects."

About the end of the year in which these papers appeared (1813) Dr. Armstrong published his work on Puerperal Fever, a work for which he always retained an author's partiality. The success of the practice which it recommended, in the particular epidemic on which it was founded, had been such as to associate its composition with gratifying recollections. In the epidemic in question, all the cases seem to have proceeded unfavorably, or even to have ended fatally, which were treated according to the opinions then prevalent, that the puerperal fever was a disease in which venesection was inadmissible. Dr. Armstrong had the merit of perceiving that the fever was combined with inflammation of the peritoneum, and that, in the early stage, free venesection and the exhibition of active purgatives were alone to be depended upon. It was observed by the critics of the day, that the purgatives appeared, according to his own statement, to have been as important to the cure as the venesection, or even of more consequence; and it is probable that bleeding was carried farther than necessary: the exhibition, also, of scruple or half-drachm doses of calomel was what, in his subsequent practice, Dr. Armstrong ceased to recommend. But it was doing no small service to practical medicine to point out, as Dr. Armstrong very forcibly did, that fever, whether puerperal or of any other kind, was generally, if not invariably, accompanied by or dependent upon some local inflammation, and that, in the early stage, free bleeding was eminently serviceable. To the somewhat unqualified manner in which Dr. Armstrong at this time expressed his opinions may, we think, be ascribed the prevalence for a certain period of an error which spread rapidly and widely,—that of disregarding or forgetting the varying type of fevers in different years or seasons, and making that practice indiscriminate which was in reality only useful or safe in certain epidemics, required to be very cautiously followed in others, and in others was fatal. All who recollect the popular opinions and practice in England with respect to fever fifteen years ago, and have observed their gradual modification, will, we think, allow that this general recommendation, for which Dr. Armstrong was in some degree accountable, was followed until experience shewed its terrible disadvantages. Practitioners thought that a new light beamed upon them. Forgetful of Sydenham, whose qualities were asserted by many eulogists to exist again in Armstrong, they were carried away by Dr. Armstrong's disapproval of the nervous doctrines of fever, by his sneers at the "terrors of timorous minds," and at the "medical phantoms, debility and malignity." They joined him in stigmatizing all who forbore from indiscriminate bleedings, in puerperal or other fevers, as "ignorant" men, inadequate to the duties of the profession. They awoke to a new belief, consolatory with respect to a disease long considered intractable, that "no species of puerperal fever was actually incurable;" and they were charmed by the boldness of an original practitioner who set at naught "grave professors," rebelled against the "doctrines of the schools," and avowed his contempt for "the speculations of the closet."

In vain did Hoffmann, nearly fourscore years ago, point out, among the *errores medicorum*, this very fault: "secundus error," (says he, in his enumeration of them,) "isque ac primus gravis, ex quo velut ex fonte complures in medicinam et praxin potissimum derivantur errores, est, quod paucissimus cognitum sit, *quid vera sit experientia medica.* Omnes fere et singuli, etiam minus periti et idiotæ, ad hanc *tanquam ad asylum* et certitudinis fulcrum confugiunt, attamen re accuratius considerata *nil minus quam experientes sunt;* siquidem genuino sensu atque conceptu experientiæ destituti, *id credunt esse experientiam* quando *semel vel bis* faustam vel infelicem in certo morbo a sumpto medicamento annotarunt efficaciam, unde mox *sibi persuadent,* inhærere adversus talem morbum sanandum *certam remedio efficaciam,* et ea propter simili in casu protinus illud adhibere *non dubitant.*"

This error of coming to general conclusions from a small number of instances, and of laying down general rules on such a narrow foundation for all future time, was never more conspicuous than in Dr. Armstrong's work on Puerperal Fever; and it was combined with a vehement desire for distinction as a teacher, or rather as a dictator of medical opinions. As an account of a particular epidemic, the treatise would have been an admirable contribution to medical knowledge: it would have shewn that there were forms of that disorder in which life could only be saved by bleeding or by free purging, or the prompt combination of both measures: but this was too limited an office for a zealous and enthusiastic mind. The character of the epidemic was pronounced to be the true and immutable character of puerperal fever; and all evidence to the contrary unceremoniously put aside. Yet such evidence, and of undeniable authority, had been advanced. It had been shewn that there were epidemics in which all or most of the patients who were bled died; others in which only small bleedings seemed admissible, and purging seemed salutary; others in which bleeding was injurious, and the use of purges of doubtful propriety; others in which bleeding had been useless, and bark serviceable. Such testimony might, one would suppose, have suggested to a reflecting physician that of these various results there might be some explanation found in the various forms of a disease incidental to the puerperal state; that, for instance, there might possibly be a puerperal fever without inflammation; that when inflammation was present, the same structure might not always be inflamed; and other possibilities. The experience of future years, and the opportunity of examining cases after death, might have cleared up these difficulties, and have laid the foundation for views of puerperal fever more correct, and the basis of more correct and rational practice. But this was left for other observers.

The general favour with which Dr. Armstrong's writings were received seems after this time to have made him a frequent contributor to the medical journals. Communications of that description are not the proper subjects of criticism; as there are few writers who have not occasionally published hasty observations in such a form. Dr. Armstrong's all bear proofs of the same peculiar impression on the author's mind, that he was a discoverer. In one we are told, *in a note*, that bleeding in the incumbent posture is less likely to produce fainting than if the patient is standing or sitting up. In another we are assured, *in italics*, that the warm bath and mercurials are often very powerful in equalizing the circulation; and a communication in the eleventh volume of the Edinburgh Journal is devoted to announcing a new view of the pathology of nervous disorders; much of which had been already, for about thirty years, before the medical world, in a paper by Dr. Parry, in the Memoirs of the Medical Society of London; but of which Dr. Armstrong appears to have had no information. This want of information affords, indeed, a treacherous facility for making discoveries. Yet the observations in the short paper now alluded to are indicative of Dr. Armstrong's constant attention to disease,—of the interest he took in it; and the enlargement of Dr. Parry's theory which it contains, or rather we should say the theory it announces is one which has since been much developed, and is pretty generally accepted; namely, that nervous affections are generally secondary, and connected with disturbed circulation, or with some functional or structural disorder in one of the three great cavities. A passage in his lecture on typhus, composed many years afterward, singularly exhibits the mental peculiarity to which we have alluded.

"It is a singular circumstance," he says, "that when I first settled in London, the current opinion among the profession was, that typhus fever originated *solely* in human contagion; and it is remarkable that it should have been reserved for me to discover that mistake in this metropolis. But the discovery, from what I before mentioned, was quite accidental, and I take no credit to myself for having made it; though, when I reflect upon it, it gives me great pleasure, because, whatever prejudice may exist in the profession, the discovery will make its way, the truth will triumph and prove useful to mankind."

If the medical profession of London did indeed, in 1818, ascribe typhous fever solely to human contagion, it was far behind the knowledge possessed by medical men in all other parts of this kingdom. The infatuation which would cherish the opposite opinion as a *discovery* would really be incredible if not so explicitly avowed. First we see Dr. Armstrong supposing the profession in London to entertain an erroneous notion, which he probably cherished himself, that typhus arose solely from human contagion; then taking up another notion, equally erroneous, that it arose solely from marsh effluvium; and proclaiming this last error a discovery, slowly making its way amidst the obstacles which envy and prejudice raised up against it, but to flourish in remote times, and reflect immortality on the original mind which conceived it.

Dr. Boott observes, in the account of Dr. Armstrong's medical opinions, appended to the biography, that those upon which Dr. Armstrong's fame will principally rest with posterity are the opinions connected with the subject of Fever, upon which he claims for his departed friend the merit of having entertained views more comprehensive, and at the same time more definite, than those generally held by the medical authorities in this country. The appearance of Dr. Armstrong's work on Tyhpus, in 1816, is thus spoken of;—

"This admirable work at once raised him to a very high eminence in his profession. It passed through there large editions in three years, and was received almost with acclamation by the medical public, not only in this country, but throughout America, where it obtained for him, from some of the most eminent professional men, the name of the modern Sydenham.

"It was characterized as a work abounding in judicious reflections, refined distinctions, and practical illustrations of the highest importance.

"In this treatise he fully demonstrated the efficacy of bloodletting in the early stage of typhus, and proved that the signs of debility and malignancy towards its close were, as in puerperal fever, in proportion to the degree and duration of the previous inflammation. He distinguished with admirable precision the different forms and stages of the disease, and established principles of practice on a rational and philosophical basis, which have for ever banished the doctrine of debility being from the first inherent in typhous fever. He substituted facts in the place of theory; restored observation to its just preeminence over preconception and conjecture; and by his clear discrimination of the different pathological characters of the varieties of the same disease, and of their distinct and appropriate treatment, has given a confidence to the mind of the practitioner which the specious simplicity of nosological definition, the false doctrine of inherent debility, and the consequent necessity of resorting to the use of stimulants, had and must ever have failed to produce. He instituted a precise but variable for an indiscriminate and exclusive practice; made opposite agents, under different circumstances, contribute to the removal of the same malady; marked with distinctness the symptoms of its varieties, the indications of their origin, progress, and termination; shewed when and how far the active resources of art against venous congestion or inflammation may be safely applied,—in what manner they must be proportioned to the existing state, and when safety alone depended upon a reliance on the unassisted resources of nature." *Memoir*, p. 17.

And again:

"He has broken and scattered the fragments of that fantastic superstructure which the inventive genius of Dr. Cullen erected, and has unwound the charm which so long fettered the minds of the disciples of Dr. John Brown. The advocates of positive debility in specific fevers can never again prevail. It is indeed a reproach upon the judgment of the profession of the last generation, that the precepts and examples of Sydenham had made so feeble an impression on their minds. What that great man failed to do, has been, in part at least, accomplished by the equally simple but more comprehensive views of Dr. Armstrong; and the honours which have so justly been paid to the memory of the one will hereafter be proportionably awarded to the other." *Med. Opinions*, v. i. p. 148.

These passages express what we believe to have been the very general opinion of the public, but more especially of the younger part of the profession, concerning the value of Dr. Armstrong's work on Fever, when it first appeared, and for some time afterward. Such also, it is sufficiently evident, was the internal impression of his own merits on the author's mind. Indeed, he could not refrain from speaking of himself in a strain somewhat similar to that of the second of the passages above quoted. He *believed* that he had banished, and for ever, some doctrine of debility; that he had been the first to substitute facts in the place of theory; that he was in short the Sydenham of his time, and, however insufficiently honoured during his life, would be looked back upon by the next generation as one who had been an almost solitary light amidst a sea of error. This agreeable conviction was the apparent result of the warm commendations poured out upon his book, for the style of the first edition is, in general, very moderate and judicious. But after this time his style gradually altered, until it became, in his lectures, discursive and faulty in a high degree. He gradually became accustomed to regard himself as a man who had discomfited the scholastic nosologists, and proved the theorists of the olden time to be mere speculators. A few years afterward we find him speaking of his innovations with pleasure, and boasting that he had "thrown off Cullen entirely." He looked back upon the practice in the Edinburgh Infirmary, save that of Hamilton, with contempt, as upon practice without principles; and he meditated a "*general* view of the nature and treatment of *acute and chronic* diseases." A somewhat voluminous writer himself, not to say prolix, his ambition was gratified by seeing his own books in the hands of every pupil, and all other reading was discouraged as a waste of time. "Some say," he observes in a letter to Dr. Boott, written in the fulness of his fame, "my lectures are not learned enough: but I shall adhere to my own observation and experience mainly; for that gives a singleness of opinion and a unity of practice which students require They may read when they become practitioners. But then, perhaps' the less the better, except matters of fact. I have never yet met with a learned physician who was a good practitioner. At the bedside such men are lost in the conflict of authorities."

Day by day were precepts like these instilled into the willing ears of Dr. Armstrong's pupils; and, whatever merits it might possess, a school more remarkable for an illiberal depreciation of learning, and for an empirical practice, and for a thoughtless attachment to the words of a master, never existed. It is therefore not only fair, but an act of justice, to students, to the older masters, and to the profession generally, to give a brief consideration of the grounds of so much assumption.

Until after the commencement of the present century, the practice in fevers seems to have been thus far defective, that all the symptoms after the first few days, or certainly after the first fortnight, were ascribed to debility. If the practitioner was called in early, he pursued an antiphlogistic plan of treatment, gave an emetic, and gentle laxatives, but seldom bled; but if late, he feared to adopt even a rigid antiphlogistic diet; and in both cases the symptoms of the subsequent stages of the fever were treated by cordials and tonics; by bark, opium, wine, and warmth. We state the truth as nearly as we can attain to it, disregarding the extravagant declamation in which it has pleased some modern writers to indulge, and their exaggeration of the faults of the old system. The writings of Dr. Currie, who proved the safety of very free cold ablutions, and the work of Dr. Hamilton, in which the advantage of free purging was made equally evident, led to important amendments in the management of all febrile disorders. Dr. Clutterbuck seems to have been one of the first of the authors of that particular period to shew the benefit of bloodletting; and the experience of Dr. Mills, of Dublin, who took a less confined view of the seat of the phlogosis so often existing in fever than Dr. Clutterbuck, completely unsettled the then established practice. British practitioners within the tropics were about the same time led to the same conclusions, and the practice of venesection was gradually adventured by them in the fevers of the south of Europe, and afterward in our own climate; in which it had been already proved by Dr. Thomas Sutton to be beneficial, in a remittent fever frequently occurring among the troops. Dr. Sutton, indeed, is to be looked upon as the first of the modern British practitioners who advocated bleeding and a rigid antiphlogistic treatment of fever; by which he succeeded in reducing the mortality in fever from one in three, or one in five, to about one in twenty. Of his treatise, which was published at Canterbury, in 1806, the reader will find an account in the thirteenth volume of the Edinburgh Medical and Surgical Journal, and we think he will agree with us, that much of the merit so enthusiastically accorded to other modern authors, including Dr. Armstrong, is really due to Dr. Sutton. The great American physician Dr. Rush had practised the evacuating system in fevers, nearly twenty years before the date of Dr. Sutton's book; and his plan is said to have been received at first, in this country, with horror and incredulity. The cold affusion was adopted in the London Fever Institution in 1805; and in 1809 the plan of bleeding, and the use of the cold affusion and purging, were already fairly established in the larger towns, at least, of the empire.

Numerous notices of the treatment of fever by bloodletting, with or without calomel, occur in the Edinburgh Medical and Surgical Journal between 1806, the year in which Dr. Sutton's book appeared, and 1816, the year in which Dr. Armstrong published the first edition of his work on fever. Such was the treatment followed and described by Dr. Palloni, at Leghorn; by Dr. Beddoes; by Dr. Jackson; by Dr. Hooper; by Mr. Boyle, in Sicily; by Dr. Clarke and Mr. Burnett; by Dr. Irvine; by Mr. Muir, at Paisley; by Mr. Parsons, at Guadaloupe; by Dr. now Sir William Burnett, in the Mediterranean; by Dr. Wilson, at Plymouth; by Dr. Mills, in Dublin; by Dr. Nicholl, at Seringapatam; and by Mr. Allan, in the West Indies. Indeed, scarcely a volume of the journal is without some evidence of the utility of bleeding in fevers, and of purging, especially by calomel. During the same period, numerous continental physicians described the fevers observed in the seat of the wars which harassed Europe, and to which some of them gave the name of "pestis bellica;" and in which the same treatment was followed. We cannot, therefore, but think that Dr. Armstrong stated the case against bleeding too strongly when he said, in 1816, that it was "still the custom of many practitioners to pour in large quantities of wine indiscriminately throughout all the stages of the genuine typhus."

It is only shewing one example taken from innumerable ones of the fluctuations of medicine, when we add that this very practice of bloodletting, with more or less of discretion, is to be found in nearly every accredited English work on fever; that it is one of the things on which Sydenham chiefly relied in some or most of the epidemics which he described; and that the method of cure which he pronounces effectual to conquer most kinds of fevers, consists of "bleeding and purging;" the very parts of practice which, during the career of Dr. Armstrong, were by some of his admirers proclaimed as the triumphs of their great preceptor over the learning and the practical ignorance of ages. But Sydenham forbore, taught by experience, from repeated bleedings. So also, in the days immediately preceding Armstrong, Parr, in the London Medical Dictionary, and Cullen, in his Practice of Physic, were careful to direct their followers to the safe employment of a means which, if carried too far, was hurtful; and we are still of opinion that some of the most judicious rules for the management of our common continued fevers will be found by careful readers in works of that period; a period, consequently, which we think it no honour, and no proof of emancipation from ignorance, to deride.

The lessons of Celsus, which embodied and expressed the practical experience of the ablest physicians of antiquity, convey the same judicious rules:—*si vires sinunt,* sanguinem mittere *optimum* est; præcipueque, *si cum dolore* febris est: this may not comprehend the whole rule, but it is clear, and safe, as far as it goes, and betrays none of the timidity, or the want of practical discernment, wherewith the followers of Dr Armstrong were so ready to charge the learned.

But it may seem to savour of pedantry that we thus go back to Celsus. The truth is, that a complete history of the method of cure in fevers would shew that few practitioners of any note have ever disregarded bleeding. Galen practised it boldly, and in various stages of fever, whenever the general symptoms rendered it desirable; and Van Swieten, who refers to Galen, approved of and followed the practice. Dr. Friend's second commentary on Hippocrates not only points out that the father of physic bled in severe fevers, but contains a case shewing that, in the early part of the last century, free bleedings were sometimes resorted to.* Dr. Mead (1751) praises bleeding in the beginning of all fevers. In 1773 we find Dr. W. Fordyce speaking strongly in favour of bleeding in fevers, and against stimulants.

Dr. Mason Good remarks of the practice of bleeding in fevers, that it has alternately lived and died away, been revived and again sunk into disrepute, for considerably upwards of three centuries; for proof of which he refers to various authors in the sixteenth century, in the seventeenth, and in the eighteenth. Let us turn, however, to still later authorities, and to general works on medicine, containing, it is presumeable, the prevalent modes of practice of the day. The rules laid down by Dr. Parr, in 1808, a year after Dr. Armstrong had taken his degree, are, as respects bleeding, no less judicious than as regards other parts of the treatment of fevers. As a *general* practice at the commencement of a fever, he stigmatizes bloodletting as highly improper. He condemns it when the patient is not in the prime of life, or when there is no topical inflammation. The reigning epidemic, the symptoms, the period of life, are, he says, to be considered. And this was said at a time when the revived treatment by bleeding was not quite assured. Of free purgatives he speaks more strongly, after Dr. Hamilton. He advises their bold employment so long as the discharges continue to be dark and offensive; but he acknowledges, what some more modern practitioners have too much forgotten, that fevers may greatly differ in different circumstances; and he should have added, not forgetful of Sydenham, (or even of Cullen, who places this consideration first in his list of circumstances which should govern bloodletting,) in differnt years, and in different seasons. If we also quote Dr. Cullen, some of our readers may think we refer to one seldom named as a practical

* Some of the English physicians, indeed, about this period, bled *profusely* in fevers: Dr. Dover, for instance; who quotes Boerhaave as following the same practice.

authority. Yet, as a practical guide, this distinguished physician is generally judicious and safe. The means of diminishing the tension and tone of the arterial system in fevers are, he says, *bloodletting and purging*. But he adds, the employment of bloodletting requires much discernment and skill; and is to be governed by a consideration of the nature of the prevailing epidemic, the remote cause, the season and climate, the degree of phlogistic diathesis present, the period of the disease, the age, vigour, and plethoric state of the patient, &c., all of which seems to bespeak the sagacious practitioner, of whom it can do no man honour to speak disparagingly. Setting aside Dr. Armstrong's protestations against the *feeble* plans of his predecessors, and looking at his own manifold cautions, we can hardly see wherein he departed from these rules of Cullen.

Our object in dwelling on this part of the labours of Dr. Armstrong is partly to vindicate some of his predecessors from unjust obloquy, and partly to enforce, what young practitioners may be usefully reminded of, this diversity of the character of different epidemics, and which demands a diverse modification or combination of remedial means. If about the beginning of the present century some of those fears of debility which have been so much the subject of scorn in later days did really prevail, and to excess, we doubt not that they arose from the visible bad effects of much depletion or rough purging in certain epidemics. Our conjecture on this particular receives strong support from that remarkable observation of John Hunter, published in 1792, that he remembered when practitioners uniformly bled in putrid fevers, but that signs of debility and want of success made them alter their practice.

The misfortune is, and the true point on which we ought to reap wisdom from these alternations of medical opinion on a very important piece of practice, that physicians have been almost always passing from one extreme to another: now lauding, and now shunning, evacuants; now dreading, and now deriding, debility. "During the greater part of the time in which I have practised medicine," says the judicious Dr. Baillie, (in 1819,) "physicians in general, and myself among that number, have, I believe, been too sparing in taking away blood in typhous fever. It was hardly ever directed to be taken away from the arm, and not often locally, except by the application of leeches to the head. Of late years, many physicians have gone into the opposite extreme, and have taken away blood too profusely. In the course of a few years, this remedy, like every other, will find its proper level." Amidst such extremes, the selection of truth is rendered difficult; but we think it must be admitted, from all past experience, varied as it is, that, in a great proportion of epidemics, many of the cases will bear, and be benefited by bleeding and other evacuations. Still, the remark of Dr. Alison,in a valuable dissertation prefixed to the Cyclopædia of Practical Medicine, is in all probability just, as regards the situation to which it particularly applies, and expresses what is always taking place in other situations,—that the fevers which prevailed between the years 1815 and 1820, (the years of Dr. Armstrong's early, but not of his best experience,) were more inflammatory, and more benefited by large bleedings, than those of recent years. To this we may very properly add, that, although a timely purgative often removes symptoms erroneously ascribed to debility, and the frequent employment of such a remedy during the course of a continued fever may even prevent the supervention of such appearances of debility, yet in some epidemics, with which most living practitioners must have had some acquaintance, and in which the intestinal mucous membrane is deeply implicated, the free use of purgatives is obviously only beneficial in certain periods of the disease. Another particular, of no small practical importance, has been observed by modern physicians: that patients labouring under fever, in their own ill-ventilated habitations, are not so well able to bear bleeding as those whose good fortune it is to be removed into the wards of a well regulated hospital. The neglect of this circumstance has, we believe, often betrayed young practitioners, and others, called upon to practise in new situations, into very unsuccessful methods of treatment. Its possibility could not have escaped Dr. Armstrong's consideration: indeed, he remarks that, in persons confined to close ill-ventilated apartments, the inflammations attendant on typhus are generally of the subacute kind. It was pointed out in the cases occurring in the London House of Recovery so early as 1812, by Dr. Bateman; and we consider the circumstance as explanatory of some difference in the experience of town and country practitioners, with respect to the capability of bearing loss of blood in fever.

We entertain not the slightest wish to depreciate the real merits of Dr. Armstrong. Perhaps there never was a more careful and observantp ractitioner, one more sincerely anxious for the knowledge which observation could teach, or more desirous of relieving his patients from the severe maladies which were the particular objects of the study. The first edition of his work on Fever only here and there betrays a slight tendency to the declamatory excitement conspicuous in his latter productions, and most so in his Lectures, the latest of his compositions. If his diligent powers of investigation had been aided by a calmer judgment; if he had come less hastily to conclusions; if he had more frequently compared his own experience with that of other observers; if he had read the older authors with more impartiality, or with more submissiveness; if he had not, in short, become inflamed with the idea of being the great reformer of medicine, he would have exhibited fewer contradictions in his opinions, and would have done much more than he ever performed for the advancement of practical medicine.

His work on Fever, although we confess it always appeared, and yet appears, to us to be rambling and immethodical, had the advantage of being the first popular production in which the new or revived practice,—the antiphlogistic practice,—was fully laid

LIBRARY OF THE

Orleans Parish Medical Society before the British reader; and Dr. Bateman, a most efficient authority in such a matter, pronounced it "one of the best treatises on Typhus that had ever appeared." His recommendation of bleeding, even to the extent to which he recommended it, was not new; but he added the recommendation of large, or what we should call excessive, doses of calomel. Full doses, as ten grains, had often been given in fever by other practitioners: Dr. Armstrong prescribed the repetition of such doses four or five times in twenty-four hours, combined with moderate doses of opium; or, avowedly following the practice of Dr. James Johnson in some tropical diseases, a single dose of a scruple or half a drachm. Such practice in fevers has, in this country, nearly fallen into complete desuetude: in fact, Dr. Armstrong himself abandoned it, as well as very free purging, except with many limitations; and thus gradually reverted to the practice of those whom he had stigmatized as haunted with fears of debility, but whose directions are not stronger concerning the danger of powerful means, when debility has come on in fever, than are his own. But he had the undoubted merit of placing the varieties of fever in a clearer light, and he described them minutely, accurately, and in intelligible language. He particularly elucidated the various degrees of congestion existing in fever; a subject on which his views are no longer considered, as they were at first rather considered to be by Dr. Bateman, hypothetical. He was unconscious, it would seem, that he was driven to admit debility as the cause of the congestion; which was hardly more than what previous authors had asserted of the typhoid form of fevers, although he limited its range within juster bounds, perhaps, than they, and avoided the mistake of considering debility as always present. The commencement of these congestive cases, without the shivering which he observed to be the precursor of fever with more excitement, led him to suppose the shivering to be connected with the excitement in the cases not congestive; a supposition that surely drew him in a line nearly parallel to the course of Cullen, which he so anxiously affected to shun. He also enforced, more impressively than had before been done, the important fact, already well known, and proved by many dissections, that, in the fatal cases of fever, inflammation was the common cause of death; and he dwelt more than others had done on the sub-acute or insidious forms of inflammation affecting various organs; applying to all inflammatory affections the division into acute, subacute, and chronic, which Corvisart had established in relation to inflammatory affections of the pericardium. In all this there was considerable merit, but not much originality.

His practical directions, generally speaking, are admirable; and his treatise found a readier way, than works recommending a similar practice had done, into the hands of country practitioners, and put an end to the custom, before discountenanced by many, of resorting to stimulants early in fever. For this not inconsiderable advantage, the public certainly appears to have been indebted to Dr. Armstrong. An opening to good practice in all the varieties of fever may, indeed, fairly be considered to have been made by his clear division of the disorder into simple, inflammatory, and congestive; divisions which seem to be based upon facts capable of verification at the bedside.

In his enumeration of the circumstances which he had observed in his subsequent attempts to ascertain the contagious or non-contagious character of typhous fever, or its communicability from one person to another, it is curious that he should omit all mention of a fact, not uncommonly observed, and which makes that contagious property, which he convinced himself had no existence, more probable than all the evidence which he adduces against his own belief: we mean the circumstance of servants leaving their places when affected with fever, and going home to their parents or friends, in whose houses fever soon after spreads; or of young persons coming to school from houses in the country, convalescent from fever, and of fever soon after affecting their bedfellows, or other schoolfellows, and of some of these being removed to their homes, and then of their brothers or sisters at home becoming similarly affected. We have witnessed such occurrences, when the distance of the places in which fever thus successively appeared excluded all probable presumption of a simultaneous malaria. We are well aware how much evidence of an opposite nature may be adduced, and how much we could ourselves adduce; but we think that the calm and full consideration of all facts of this kind should have led to the conviction that there were yet some circumstances unknown or unexplained, which might eventually reconcile apparent contradictions. Such consideration, it really appears to us, was what Dr. Armstrong was latterly rendered incapable of, partly from his excitable constitution, and partly from the unconscious bias given to his opinions, by his feelings respecting those who had taken different views. His admirers may feel reluctant to allow this explanation: but, if we reject it, it will be difficult to give Dr. Armstrong credit for that candour which we are not disposed to deny that he possessed, and that love of truth by which we really believe him to have been animated. Another explanation offers itself, in his unwillingness to admit for continued fever, in any circumstances, what he could not claim for remittent fever or intermittent fever; and one of the discoveries on which he considered his fame to rest was, that these were all modifications of one disease; a view, however, which had been taken by previous writers. The variety which he admitted to exist in these three modifications might have obviated, one would think, any invincible unwillingness to admit that the severest form of the three occasionally became a communicable disease; or even to admit the bare possibility of the same occasional communicability in the other forms,—that is, in the remittent, and even in the intermittent, forms of fever. This,

CHARITY HOSPITAL, MEDICAL LIBRARY

we apprehend, would have been the philosophical method of investigating so great a question,—a question which we can by no means consider as yet set at rest.

Dr. Armstrong's descriptions of disease are faithful and graphic; and cannot be read, together with his observations on the treatment without an impression on the reader's mind that he must have been a most watchful and excellent practitioner. If this was much doubted by his contemporaries, we are inclined to ascribe it to those declamatory habits which we shall find it our duty more particularly to speak of; and which led his followers to extremes that his own practical acuteness caused him to avoid; and reflected upon him that undeserved censure to which his kind biographer alludes (vol. I. p. 212,) of having been the "indiscriminate advocate of depletion." We think also that we see, in his directions for the treatment of a severe form of congestive fever, sufficient reason to doubt the general success of the plan he laid down for imitation. When "the extremities are cold, the respiration laborious, the pulse heavy and oppressed," we may be allowed to question the advantage to be derived from bloodletting, as a general measure; and the caution with which Dr. Armstrong surrounds this direction, and the allusion to the possible diminution of heat, increased weakness of pulse, more embarrassed breathing, and greater debility, clearly indicate those cases which, in his own practice at the Fever Hospital, were, we know, considered unfortunate illustration of his views; and point to the source of much of the bad practice in fevers which was to be met with, in this island, about twelve or fifteen years ago. The practical error apparently arose out of too strong an attachment to his own theories; and, together with his apparent disregard of the diversities of epidemics, constituted the most serious of his faults as a practitioner and a teacher. His directions, also, respecting the administration of wine in certain stages of fever are loose and unsatisfactory, and not to be compared to the more exact rules laid down by Dr. Bateman. With these exceptions, his practical observations on fever are admirable, and such as his indiscreet followers too little attended to. It is impossible not to admire the indefatigable industry with which he prosecuted this subject to the end of his life, and when the practice in which he had become engaged was considerable enough to have excused him if he had desisted from all other labours.

Dr. Armstrong was encouraged by the successful appearance of the first edition of this work on typhus, to quit Sunderland, and to settle in London; where his first feeling, very natural to one neither rich in money or London connexions, are thus affectingly described by Dr. Boott.

"In October 1817 he resigned his situation as physician to the Sunderland Dispensary; and in February 1818, after placing his wife and his two children in lodgings in Durham, he repaired to London, with no other recommendation than that which his works and reputation afforded him.

"He took lodgings at No. 38, Great James street, Bedford row, where he resided several months alone. This was the most trying period of his life. All those domestic sympathies upon which he so much depended for happiness were far removed from him, and he felt as it were alone in the world, anxious about his present and uncertain of his future fortunes. He never, to the close of his life, courted general society, and had few inducements to mix in public amusements; for his tastes centered in his professional pursuits, and his enjoyments in the bosom of his family, and in the familiar society of a few personal friends. His sensibilities were acute, and his mind simple and discerning in its instincts and desires. He had left a society to which he was attached by the ties of gratitude; and in the oppressive solitude of his present situation he keenly felt the loss of his early friends, and became fully sensible of the hazard to which he had exposed the interests of his family. He has often told me that the loneliness of his situation at times overpowered him; and that so oppressive was the busy scene around him, in which he stood a stranger, uncared for and unknown, that he sometimes found relief in tears, and tried to drown the consciousness of sorrow, by seeking sleep in his darkened chamber at noon."

Within a year from this time, his prospects of practice were very much improved. He published his opinions on various practical subjects;—on Measles, Scarlet Fever, Consumption, Chronic Diseases, Sulphureous Waters, External and Internal Inflammation, Insanity, &c.—and his plain and unpretending manners secured him many friends. His fondness for his profession led him in his conversation, as in his writings, to dwell minutely upon common points of practice, on which, without offending the prejudices or alarming the pride of his hearers, he became a great authority; and he possessed what we believe is always found to be a substantial recommendation to success in medicine, an almost exclusive attachment to it. There was this peculiarity about his mode of expressing himself, that his earnest dwelling even upon matters taught in every elementary book, made the hearer believe the ideas were as original as he sincerely appeared to consider them. There are peculiarities in his work on Scarlet Fever, Measles, &c. which exhibit the source of this complacent dwelling on ideas which he conceived had not yet reached others, because they had only recently reached himself. His very preface shews that the sedateness of mind maintained throughout his treatise on Fever, had been disturbed by the praise he had received. He begins by declaring that *names* have governed or influenced the world in almost every age, and that an æra has arrived in the history of physic, when the vague conjectures of the most celebrated individuals must give place to the inferences of unbiassed observation. He speaks of the improvement of the medical art as one of the most important of passing circumstances, which will strike those who look back upon the actual time in which he was writing. He declares that experience and reason have gained a *signal triumph* over the *dogmas of the schools* and the prescriptions of speculative authorities. "Once," he says, "our libraries were crowded with Commentaries on the opinions of others, a state of literature which, when general, always marks the declining genius of a country; but now, instead of such servile employment, the press daily teems with the productions of those who possess that independence which prompts them to observe and to think for themselves." The preface containing these expressions is dated in November 1818, when Dr. Armstrong had begun to lecture in the Borough, and they are so plainly meant to refer to himself that we cannot help thinking they admit of explanation by a circumstance which we shall presently have to mention. The work to which they are prefixed is one which contains many practical observations of utility, that might most advantageously have been compressed into half the space they occupy; and the pages exhibit a few indications of that desire to be thought learned to which we have already alluded, and which was hardly consistent in so warm a contemner of "the schools." Among these are frequent and unnecessary references to works on general subjects. When Dr. Armstrong wishes to tell the reader that if skill is shewn in curing a distemper, wisdom is shewn in preventing it, he refers to William Penn's Fruits of Solitude in support of this not very paradoxical observation, and particularly directs the reader to "see a new edition, p. 52, London: printed for James Phillips, George yard, Lombard street;" this new edition being at that time five and twenty years old. When he would praise that book on the Constitutional Origin of Local Diseases, which Mr. Abernethy was wont to recommend to his own patients, he does so "in the language of Bacon." The obvious truth that debility disposes to disease, is asserted to be "as old as Celsus," and Celsus is referred to "excudebat Gulielmus Bell." The amenity of the ancient climate of this island is supported by a reference to a certain "History of the Decline and Fall of the Roman Empire, by Edward Gibbon, Esq. *of Dublin:* printed for Luke White, No. 86, Dame street." These trifles would not merit notice but for the derision with which Dr. Armstrong so often spoke of all learning.

A physician who removes from the provinces to London is like a man plunged into a deep lake, and is some time before he again rises to a level with visible things. It is mortifying, no doubt, to find the merits so cheerfully acknowledged in the country, lost amidst the more active talent and perhaps the wider range of acquirement of the metropolis; and without a good deal of confidence in a man's self, and certain pecuniary resources, the first years of a life so spent in London must necessarily be years of much anxiety. Within the sphere of our own acquaintance or reading, we can find no example of a rise so rapid as that of Dr. Armstrong from comparative obscurity to a large practice. He was already a distinguished London physician when he presented himself, as all graduates of other universities than Oxford and Cambridge are under the necessity of doing on attempting to practise in London, to the College of Physicians for examination and license to prescribe. Much to his own vexation, and not a little to the surprise of his admirers, he was rejected by the college. Dr. Boott thus speaks of this remarkable incident in the life of his friend.

"He had, perhaps, undervalued the estimate which the Board of Examiners place on classical diction, and the alphabet of the profession; for this distinguished physician, who had received a diploma from the most efficient and most celebrated school of medicine in Great Britain, who had been in successful practice eleven years, and was the author of three of the most popular works which the medical press of this country had ever put forth, the fame of which was still sounding in the periodical journals of the day,—was rejected as incompetent to continue in the practice of his profession in London, and as undeserving the honour of having his name enrolled among the members of the College.

"This public stigma, of the justice and motives of which I leave others to judge, was not without its natural and perhaps salutary effects upon the sensitive mind of Dr. Armstrong. His nature was mild, but too dignified to submit to insult and unmerited wrong, which threatened injury to his own reputation, and ruin to the welfare of his family. He did not admit the necessity of any particular attention to his profession to qualify him for passing the usual examination the next year, as he was aware that the first rejection was generally the only one. But he felt roused to the due assertion of his own claims to respect; and from the impressions which this act of wanton power made upon him are to be ascribed much of that indignant tone which afterwards sounded in his lectures on scholastic institutions." *Memoir*, p. 30.

The evident impossibility of so respected a physician being ignorant of the practical part of his profession, added to the popular ignorance of the nature of the examinations at the college, caused this rejection to give Dr. Armstrong all the advantages of martyrdom. The governors of the Fever Institution, having a just reliance on his abilities and his humanity, rescinded a law which would have been fatal to his claims to be their physician, and elected him with a kind of triumph. The public looked upon him as a persecuted man. Many suspected the College of jealousy of his rising celebrity. The feelings of his pupils were often subsequently appealed to; the decision of their juvenile judgments was unanimously in favour of the popular teacher; and they were taught to speak contemptuously of colleges, and learning, and Latin. Yet to suppose that the examiners of the college would have rejected a physician already distinguished unless his answers had been peculiarly unsatisfactory, would be an absurdity. No dispassionate person can doubt that they must have been *compelled* to the rejection by some unwonted deficiency: and such deficiency, we apprehend, must have been made too glaring to be passed over. And although a man may practise physic and write upon it who has forgotten much of his anatomy, and almost all his Latin, we do not well see, so long as colleges affect to patronise either anatomical science or liberal learning, how they can consistently pass over an almost total ignorance of both. We presume there is not a physician to be found, however unpleasantly he may reflect on the politeness with which he was converted for a certain consideration into a licentiate, who will not acknowledge that his examination at the London college was at least sufficiently elementary and sufficiently courteous. We believe also that some classical acquirement is generally accepted and taken into the account by the examiners. The very eminence already attained by Dr. Armstrong must have prevented his being rejected on slight grounds, and have caused his examiners much to regret being obliged to incur the odium of his rejection. In short, nothing would ever have convinced us of the injustice of Dr. Armstrong's rejection, except the publication of the questions and answers; and this he never proceeded to.

Two important questions here present themselves, in which the honour of the examiners is somewhat concerned; whether, in the first place, the College of Physicians has not occasionally admitted men fully as unqualified in certain points as Dr. Armstrong? and, secondly, how had Dr. Armstrong himself contrived to pass the former ordeal of Edinburgh? As regards the licentiates, we think we could point out one or two whose Latin, unless they were inspired for the occasion, must have discomposed the dignified gravity of the learned president himself; but then it is to be presumed they were not so oblivious of their anatomy as to be caught in any of the common traps set in desperate cases to catch the unwary. The Edinburgh college demanded a very moderate share of Latin. It used even to be thought not quite safe, or quite etiquette, to speak it too well. Then in ten years Robertson's *Colloquia* had oozed out of the palms of the hands, and were not perhaps producible in London. Besides this, there was a very proper consideration, we believe, in Edinburgh, for those students who had been seen to be diligent throughout their curriculum; whose means were scanty; and to whom rejection would have brought ruin and despair. Thus, although an accomplished student went through a very difficult and comprehensive examination in Edinburgh, the less efficient candidate was let through more easily; and none were rejected save the notorious indolent or the notoriously dissipated. Anatomy, too, was not one of the things for which Edinburgh was most famous. So that in ten years a graduate of Edinburgh, although that ten years might have made him a good practical physician, might very possibly have become defective in anatomy, and very far from eloquent in Latin. What the value of any such examinations was or is, let others judge. With the public it is well known that they avail nothing.

At all events, Dr. Armstrong's rejection did him no mischief, except in so far as it strengthened all his prejudices, and suggested divers indignant apostrophes touching academic learning, for the lectures of future years. Neither did it add to the authority of the college; as it afforded an opportunity to a portion of the public of setting it at naught, and dispersed the ideal supremacy which previously hedged it.

To us, however, it always appeared that there was both impropriety and puerility in the observations which Dr. Armstrong addressed to the young men attending his lectures when this unhappy recollection arose in his mind. Thus when, examining the origin of typhus, he says, many men believe it to be contagious "because they have been told so *at school or college*, precisely on the same principle that children take the assertions *of their fathers and mothers upon all subjects.* Now, as our fathers and forefathers of physic have often been mistaken," &c. And again, in the next paragraph, "the question whether typhus fever is contagious or is not contagious, cannot be decided *by any reference to black-lettered books;*" &c. All of which seems very flippant and pointless; for most men have a better reason for believing or disbelieving in contagion than having learnt so at school or college; not many children believe all that their fathers and mothers tell them; and we question whether even the oldest fellow of the college would seek for proofs of it in black letter. But all this delighted the young students. And when the lecturer wound up by an appeal to the sober judgment of those whom he was there to teach, exclaiming "Now, *I ask you once more,* how such things could by possibility happen if typhus were that contagious affection which *schools and colleges,* and which those *secondary intellects* who borrow their notions wholly from such authorities, would have us implicitly believe?" —the lecture room rang with acclamations, and the excited teacher felt like one before whose discoveries all old things were passing away.

As we have very unreservedly noticed these and other circumstances, but yet wholly without the wish to give an unfavourable colour to them, we think it but just to allow Dr. Boott to explain the causes which in his opinion led Dr. Armstrong to the eminent success which he obtained in London. He is speaking of the period immediately subsequent to Dr. Armstrong's appointment to the Fever Hospital.

"He was now encouraged to indulge in favourable views of his future prospects. His family had joined him from the country, and he removed to a house in Southampton row. The celebrity of his writings had introduced him into some practice among a few members of the profession who had been impressed through them with a confidence in his talents; and the reputation he had acquired on the subject of fever, together with his early appointment to the Hospital of St. Pancras, soon led him to be considered as the highest authority upon typhus, a pre-eminence which he maintained to the close of his life. This general confidence in his experience and abilities was one of the most honourable and prolific sources of his prosperity, and it was shewn in the frequency of his being consulted by his medical brethren, in their own illness, or in that of the members of their family. It has been said, and I believe with truth, that, during the eleven years he resided in London, he was called upon to attend more medical men than any other member of the profession. He owed his success in London to two causes, for no one had ever fewer adventitious aids to success, and the one reflected as much honour upon his talents as the other did upon his disposition. Those members of the general profession who had once experienced the benefit of his counsel and assistance, could seldom be induced to recommend any other physician, so strongly impressed were they with the simplicity, the originality and success of his views and practice: and those families who had once had an opportunity of feeling the effects of the gentleness and delicacy of his manner, could think of no other adviser. There are many persons in and out of the profession who will admit the truth of these remarks, and who will confess that the loss of this eminent man appeared to them irreparable. He had the faculty of communicating his ideas to others in the most easy and intelligible manner, and, from the fertile resources of his own mind, of throwing light upon the most obscure and involved cases. Those difficulties which embarrassed common minds seemed at once charmed away by the magic influence of his own; and his opinions were delivered with so much candour and perspicuity, that while others bowed before the superiority of his intelligence, they were instinctively impelled to place the fullest confidence in his skill and integrity, and to feel an irresistible affection for his character as the man, blending with their admiration of his talents as the physician. His manners were simple almost to a fault, and were at first forbidding, from the absence of every thing like an attempt at effect; but no sooner did he enter upon the consideration of a case, than it was apparent he was completely absorbed by it. His seeming reserve at once gave way to a visible feeling of deep and tender interest in the welfare of his patient, who felt satisfied that he was in the hands of an amiable and a sagacious man, to whom he might confidently intrust himself." *Memoir*, p. 32.

These highly favourable representations of the impression produced by Dr. Armstrong in private practice are supported by some interesting anecdotes, and, whatever may be thought of his originality or judgment as an author, no doubt can remain on the reader's mind that he was a most attentive and sagacious practitioner. In the years 1820 and 1821, Dr. Boott attended his practice in the Fever Hospital, and he bears strong and, we doubt not, just testimony to Dr. Armstrong's conscientious discharge of his duty in that admirable establishment; to his humanity, urbanity, and punctuality. No situation could be more interesting to a physician whose attention had already been so much occupied with fever, and the daily experience of such an institution would at once shew him the limits of his former experience, and the many yet undescribed varieties of continued fever. In 1821, Dr. Armstrong first became a lecturer, in the school established by the late distinguished Mr. Grainger; and his fluency, animation, and the general kindness of his manners, soon ensured him a high degree of popularity among the students. Of his possession of all the higher qualities of a lecturer, his biographer speaks in language approaching to enthusiasm. We feel it incumbent upon us to quote a part of his description :—

"He united in his mind, with the elements of a sound judgment, two opposite and often irreconcilable powers; that of discerning particulars and of combining them together, so as to see in one glance their origin, connexion, and effects. He thus acquired practical knowledge from his observation of the phenomena of disease, which he freely communicated to others from the learned and well arranged volume of his memory, and he was possessed of an intrepidity of character which made him fearlessly assert whatever he felt to be true. Though of a mild and pliant nature in the hallowed seclusion of domestic society, he was, as a public teacher, loud in his denunciations of what he considered to be erroneous in the maxims and practice of his profession; and if at times he seemed to sound too indignant a tone of remonstrance against the prejudices of the schools, much of the fervour of his language and feelings is to be ascribed to the indignation which he felt at the conduct of one of these bodies towards himself.

"The effect his lectures produced was electric. The energy of his manner, the fine intonations of his voice, the facility and correctness of his diction, the strain of impassioned eloquence which often burst from him, riveted the attention, and made even those who could not entirely adopt or appreciate his opinions, sensible that he was uttering the deep convictions of his mind; and there was so much of chaste and often of pathetic feeling, so much of the refined sensibilities of his nature blended with his discourse, that those who were compelled to admire his talents felt confidence in his virtues; and while they revered the Professor, they loved the man." *Memoir*, p. 58.

Such praise convinces us more strongly of Dr. Boott's admiration of Dr. Armstrong, than of Dr. Armstrong's possessing exactly the qualities desirable in a teacher of practical medicine. It impresses us with a belief of the irrelevancy of the topics, and the unrestrained declamation, which we have often been told were occasionally touch upon and indulged in; and when Dr. Boott adds that Dr. Armstrong's lectures "were not pronounced in the formal diction of arbitrary and systematic terms," but were "the views of a master-mind, which borrowed no aid from without;" and when he says that Dr. Armstrong "divested the science of medicine of the meretricious allurements it had long worn, and dressed in artless guise;" and that he was "deeply impressed with the defects of the scholastic mode of education which he had early acquired;" our thoughts cannot but again revert to the noble simplicity, the profound and elegant learning, the unaffected originality, and the manly and appropriate eloquence of him who was the teacher of medicine in Edinburgh when Armstrong was a student—and we must confess that Dr. Armstrong does not gain by the comparison with one whose scholastic method he so unhesitatingly condemned.

It is not that we are insensible to the peculiar merits of Dr. Armstrong's lectures, as exemplified in the spirited transcript of them edited by Mr. Rix, and the fidelity of which is unquestionable.

We admire, in almost every page, the precise and cautious practical directions; the striking allusions to instructive cases; the urgent recommendations of the pupils to be careful, to be diligent in observation, to avoid hurry and heedlessness, to be attentive to the poor. Nothing can be more excellent than the rules laid down for all the parts of the delicate management of fever patients; nothing more judicious than the general instructions arising out of the lecturer's perfect knowledge of mankind, and his perfect discrimination of the relative characteristics of the upper, the middle, and the poorer classes. His prudent admonitions respecting the employment of some of the heroic remedies, as mercury, arsenic, and colchicum, attest his powers of observation and his practical merits. We do not quarrel even with a few eccentricities, such as a depreciation of what are called saline medicines, which are so universally refreshing to patients in febrile disorders; and an unaccountable prejudice against the term *constitutional*, to which Dr. Armstrong was unable or unwilling to attach any definite meaning. But there is still much, very much, in the lectures, of which we cannot but most strongly disapprove. There is a frequent affectation of simplicity, or of what Dr. Boott has indulgently called dressing medicine in artless guise; of which the rejection of the term pericardium, and the substitution of the inelegance of "bag of the heart" may be taken as an example. This was done, no doubt, to avoid a word derived from two Greek words, taught "in schools and colleges." Yet to avoid this learning, some inaccuracy was incurred. The fantasy of putting down ancient learning, indeed, leads Dr. Armstrong into continual rhapsodies, of small benefit to his students, and sometimes (to us at least) quite incomprehensible. Would any one believe, for instance, that the following passage is part of thirty lines of a like description in a section of not more than forty-two lines, devoted to the diagnosis of cystitis from hysteritis.

"Foresight is second sight. No species of knowledge is so important as that foresight of practitioners which enables them to prevent diseases. But another kind of foresight which a medical man should daily endeavour to acquire, consists of a power of foretelling events; a power equal to that which our poet gives to the wizard, who exclaims—

> 'Tis the sunset of life gives me mystical lore,
> And coming events cast their shadows before:'—

it is allowable in a poet to speak of 'mystical lore,' but it will not do in physic. We have enough of art and mystery and so on in an act of Parliament; but common sense despises and rejects all the mummery which exists about art and mystery in schools and colleges and articles of apprenticeship. No species of human knowledge is mystical: we ought to have no mystical lore; for knowledge is only mystical in order to conceal something wrong. Johnson has said, "No man can be great without labour;" &c.

We do not know what the young gentlemen in the Borough thought of these fine sentences, but if this be common sense, give us the despised mummery of schools and colleges.

Our readers might think it an ill-natured task if we were to dwell upon the innumerable quotations introduced into the lectures, from every book, apparently, that Dr. Armstrong ever read in his life; and we shall only remark that they are generally useless and inapplicable to his subject, and sometimes not understood by himself; as must always happen when a lecturer is too anxious to shew his reading. How inconsistent does such anxiety appear with a professed contempt for learning! Not less inconsistent are Dr. Armstrong's frequent denunciations of popular physicians, and of men in large practice; when his own heart was swelling with desire for that very popularity and that very practice.

Unless we entirely mistake the feeling which exists throughout our liberal profession, there are not four names more honoured than those of Mead, of Heberden, of Cullen, and of Baillie. Extensive acquirements, princely liberality, the most enlarged philanthropy, the utmost practical knowledge, were in different degrees united in these distinguished men, and to mention them makes every lover of his profession love it the more. For these great names, however, amidst many declamatory passages, Dr. Armstrong could not spare a word of praise. Whilst he allowed himself to be carried away from his subject in praise of General Washington or Miss Edgeworth, or the author of Waverley, he can only allude to Mead as "a learned man and an honest man, though not a man of much talent, for men of original minds never waste their time in perusing books of old times." Heberden, he says, was "a very superficial observer of nature," an opinion which could not easily be matched for its injustice. Again, "Heberden was a very popular physician in London; but his literary productions will soon be forgotten"—and then, with a fine flourish,—"they will be swept away by time, as wrecks from the shore by a springtide." Surely Dr. Armstrong had never read Heberden's Commentaries; or perhaps the circumstance of their being written in Latin prepossessed him against a writer, of whom, if it had only been for his minute and accurate observation of disease at the bedside, Dr. Armstrong ought to have spoken with more respect.

Dr. Baillie fares a little better; his quiet demeanour in a sick room is mentioned in terms of commendation, and his moral character extolled; but his practical merits are entirely passed over. It is, however, upon the devoted head of Cullen that Dr. Armstrong pours out all his sarcasm and all his contempt;—upon Cullen, the clearness, fidelity, and elegance of whose descriptions of disease have attracted the admiration of scholars, and won the grateful praise of some of the most eminent physicians of the last eighty years. If Dr. Armstrong mentions an instance in which he himself made a mistake, he adds, "this was the consequence of my reliance on the accuracy of Cullen's definitions. I beg therefore that you will be upon your guard against all big-wigs," &c. &c. In another place he indulges his humour after this fashion:

"You may have seen a dog in a wheel turning a spit on which a leg of mutton is roasting. Now, the nosological practitioners and writers exactly resemble this dog. No one has done so much mischief with respect to the profession of physic as Cullen has; and it is my amazement to know that his absurd system yet obtains in medical examinations. No man at all versed in modern pathology attends to such a system of crudities and errors. It obtains only in the shades of men's closets, or in the cloistered walls of schools and colleges. But the rising generation will, I trust, put an end to all such lingo and legerdemain."

It would be difficult, in the whole range of medical vituperation, to adduce more groundless accusations, couched in more inelegant language? What could be expected of young medical men, accustomed to hear such ungenerous criticism from the lips of their teacher? what but self-conceit the most ludicrous, based upon ignorance the most profound?

Perhaps we look back with too much respect to Edinburgh. We well remember the late Dr. Duncan telling us, in his careful clinical lessons, how best to investigate the cases of our patients; and, alluding to their loquacity, counselling us to let them talk without too much interruption, to hear them patiently, and to question them minutely, gathering the essential facts as best we could. Dr. Armstrong, who despises these scholastic phrases, speaks with more vivacity. "If any of you," he says to his admiring pupils, "be tired of my minuteness, and wish to practise medicine boldly, the best way is to carry a plaster about with you, in your waistcoat pocket, and, whenever a patient is complaining, you have nothing to do, but, without asking any questions, to clap this plaster on the ailing part, and prescribe fried eggs and bacon." We have heard something of, and read something from, the Gregories of Edinburgh, on the qualifications of a physician; but Dr. Armstrong defines them with more originality. To make a medical man, he observes, requires, "in the second place, experience: not such experience as a dog gets in a wheel by turning a spit to roast a leg of mutton, nor such experience as a horse gets by following the same daily round in a mill; but experience which is," &c. We had idly imagined that there *was* such a thing as a gouty *diathesis*, or a disposition in certain constitutions to gouty forms of inflammation: Dr. Armstrong not only doubts this, which he had a perfect right to do, but thus expresses his doubts to the ingenuous youths around him: "But what is this something?" *(gouty diathesis.)* "Is it a goblin dancing about the body? It is a phantom, a medical phantom, which haunts schools and colleges," *(still schools and colleges!)* "and, entering the closets of big-wigs, plays all sorts of pranks in their imaginations. It is mere nonsense; stuff, such as dreams are made of:" thus closing a piece of unmeaning declamation with an unapt quotation, and all in triumph over schools and colleges. "I never met," he says in another lecture, "with a learned physician—I mean a man of black-letter learning," (still *black-letter!*) "who did study the phenomena of nature." And again, "I will hack and hew this great tree of nosology, till I bring trunk and branch to the earth." "Look," he says, "into the most popular work on the subject,"—and to what work does the reader think the hearer was referred?—"look to the work of *Dr. Thomas!* It is any thing, every thing, or nothing!" Dr. Armstrong not only declares that "the symptoms, pathology, and practice laid down in that book are current in this country;" but, lapsing into his favourite notions, "current in our schools and colleges;" and he winds up this extraordinary charge with Pope's couplet in allusion to the insincerity of Addison.

But we arrest our pen. We leave unquoted scores of passages equally exceptionable, and exhibiting every variety of fault that could beset a teacher, from vapid common-place to something very much like raving: as, for instance, when Dr. Armstrong leaps from contagion to the holy alliance. We feel it an imperative duty to notice such harmful, vicious modes of affecting the inexperienced minds of students, and of protesting against their being held up for imitation, or as worthy to be admired. It is not by pandering to the ignorance and prejudice of half-taught young men, that any medical school, or any teacher, can obtain a permanent celebrity; and we do but justice to the most eminent living lecturers of London, when we say that we believe none of them follow such methods, or feel that lively horror of academical modes of education, or of schools and colleges, or even of black-letter, which Dr. Armstrong had cherished until it seems to have become a disease.

In four years from the commencement of his lectures, Dr. Armstrong had succeeded in obtaining a great share of practice. "I had left him," says Dr. Boott, "in 1821, struggling into notice, but still in some degree doubtful of the propriety of the step he had taken in removing to London; and, on my return from Paris, in the summer of 1825, I found him in the full enjoyment of fame and prosperity." In 1822 he had printed a paper in the Medical Intelligencer, conducted by his friend Mr. Haden, "On the Origin, Nature, and Prevention of Typhus Fever," in which he materially modified his original views; and another "On the Utility of Opium in certain Inflammatory Disorders;-the object of which was to shew its utility, in free doses, in acute and subacute inflammatory diseases. The employment of smaller doses of opium conjoined with calomel had been previously recommended by Dr. Hamilton, of Lynn, and followed with much advantage, and more generally, we think, than the plan of Dr. Armstrong has been.

In 1826, Dr. Armstrong began to lecture in Little Dean street, Soho, continuing also his lectures in the Borough; but this double occupation was abandoned in 1827, and his labours were then wholly devoted to his increasing practice, and to lecturing in his original Borough School. Indeed, his attempting the singular labour of lecturing twice a day, in different and remote schools, was far from prudent, especially as he had not gone thus far in his ardent and laborious career without some warnings that the machine was going too fast. Three years previously, after an attack of dysentery, he remained for some time (he says, in one of his letters to Dr. Boott,) affected with a daily dread of sudden death, and had one or two severe attacks of giddiness. He speaks, in his lectures, of habitual evening fever supervening on slight excitements.

Until December, 1828, however, he went on in a constant course of exertion; visiting numerous patients, and each day exhausted by his occupations. Still he was desirous of publishing a long projected work on Chronic Diseases, and he completed some fasciculi of Morbid Anatomy of the Stomach, Bowels, and Liver; a work of which the publication was commenced amidst the pressure of other engagements, and at length interrupted by his failing health.

The premature conclusion of this active and useful life is truly melancholy. We contemplate the gradual elevation of an aspiring man with interest; and, when we have watched him to the summit of the steep where fame's proud temple shines, he vanishes like a dream. To be born; to live some years of youth in ignorance; to feel the mind lighted up with knowledge and warmed by ambition, as manhood approaches; to enter on the great struggle for existence; to succeed partially; to see afar off the truths which life is too short to allow us to reach; to lay schemes, all unmindful of this narrow term, and in the midst of these schemes to die;—of how many medical men has such been the sad compendious history! Of all the struggles and pains which afflicted them, of all the hopes by which they were cheered; the dreams they cherished, and the fears which haunted them; nothing remains when death has removed them. The whole of life, which once appeared a voluminous scroll, is compressed into a short record of what good was pursued, and what useful ends were effected.

Dr. Boott has, we feel assured, very faithfully given us a description of the opinion he was enabled to form of Dr. Armstrong, in consequence of the intimate friendship subsisting between them. But he has either thought it a part of a biographer's office to conceal every trait not positively deserving of praise, and thus to compose rather an eulogium than a biography, or he was unacquainted with peculiarities which to others were well known, and which help to illustrate a character apparently not easy to be understood, and concerning which very opposite opinions prevailed. By those who were in habits of intimacy with Dr. Armstrong in his latter years, when he had surmounted the difficulties which have overwhelmed so many aspirants for professional success, and when his receipts were very considerable, it was perceived with sorrowful surprise that the smallest deficiency in the amount received in any one month made him prone to despond, and to indulge in a morbid apprehension that he was destined to be the victim of extensive professional jealousy. A professed, and we believe a sincere reformer, he beheld with little pleasure new schools arising in which new methods of teaching were to be followed, and he prognosticated their failure. The lectures of men many years his juniors, and who never affected to rival his fame, gave him an uneasiness which he could not refrain from exhibiting. He was still governed by an ambition of which it must be allowed that the objects were by no means unworthy, but which was in itself too restless for his own happiness. Even when the disease which proved fatal to him had begun to shew itself in his appearance and countenance, his mind was busy with the plans of future years. He meditated a removal to the west end of the town, and, in about ten years more, he considered that he should probably be rich enough to seek a seat in parliament, where he avowed his intention of proposing some of the medical reforms which no one then dreamed would be sooner accomplished. The tranquillity of his manner whilst detailing these plans was remarkably in contrast with the scope of his aspirations; and, now that death has interrupted all his earthly hopes, a vivid impression of his appearance and conversation remains in the recollection of those who heard him, which nothing can efface. Yet this continual desire to be the first man in his profession, the impatience it created of the obstacles which arose out of his deficient primary education, the groundless but tormenting ideas which always seem to have haunted him of the uncertain tenure of his practice and his popularity, and his extreme openness to every exciting impression, little fitted him for the daily toils and collisions of such a whirlpool of intellectual struggles as London. With a more patient temperament, and in a more limited scene, and with a more chastened ambition, his useful labours might perhaps have been calmly reviewed by himself, and even prolonged many years.

Among the singular opinions which he had adopted was one opposed to the general and well-founded notion prevailing in this climate, that free exposure to cold air when the body is in a state of inaction exposes to the chances of cold and cough. This notion Dr. Armstrong considered as a mere prejudice, and fit to be exploded. We were aware of this peculiarity; and we observe that Dr. Boott mentions exposure to the air in all weathers in his carriage with the windows down, and leaving off flannel, among the suspected causes of a cough which first excited any uneasiness in the minds of his friends. Physicians are so prone to fancies of this kind that one cannot wonder to find unprofessional people duped by the most extravagant assertions of pretenders: but surely it is a reproach to medical men to be much divided respecting the common influences of external objects on human health. With some interruptions, he continued to lecture; and, with the usual infatuation of the consumptive, felt no anxiety either on account of his cough or his debility; early and warning symptoms, which his acute observation would not have passed over in any of his patients. The remainder of his life was but a continual struggle between an ardent and unyielding mind and a remorseless malady. From occasional excursions into the country he generally returned refreshed, and desirous of resuming all the duties which he was again and again compelled to relinquish, until he at length felt that he must wholly desist from them. Often as we observe medical men exhibiting the same self-delusion as others, when actually sinking under phthisis pulmonalis, suspicions, one would suppose, must now and then present themselves, that all life's scenes are drawing to a termination. It is difficult, before age has brought some indifference to worldly pursuits, to accept the signal which shews us that we must retire: every hope, however frail, is willingly received,—every little amendment renews those sanguine expectations of returning health, and strength, and activity, which are never to be realised. Restless nights, languid days, feverish evenings, hours unoccupied, a wasting frame, the intermission of all pursuits, even these preludes to more complete death, do not seem to interpose themselves strongly between the patient's hopes and the busy world, with which all but himself plainly see that he has done for ever.

After several retreats into the country, however, Dr. Armstrong for a time lost hope, and for the first time expressed an apprehension that he was consumptive: but the country air again revived his spirits, and he again felt assured that he should recover. This was in May, 1829. A single week in town brought back his debility; and it was with great reluctance that he left his summer course of lectures unfinished, devolving the task of its completion on Dr. Boott. Two days spent at Richmond dissipated the gloom that had been gathering round him, and he proceeded to Worthing with cheerful expectations, which, whilst able to enjoy the refreshing breezes from the sea, he for awhile continued to indulge. He then began to believe that his complaint was chronic pluerisy; and nothing was more unwelcome to him than were the visits of his medical friends. He represented that their calls excited him: probably he perceived that their words or their countenances were opposed to the wishes he was so unwilling to abandon. He confessed, indeed, after a visit paid him by Dr. Boott and Dr. Clark, that he was quite satisfied they both thought his case hopeless, and saw through their evasions of his questions on that point; but he added, that such a conclusion was by no means warranted by the symptoms.

Finding the sea-air too keen for him, he removed to the neigh bourhood of Dorking, early in August. Daily rides and walks in a beautiful country so animated and revived him that, in September, he reported himself "better, and determined to be quite well this day five weeks." Within a fortnight, when seen by Dr. Boott, he spoke of his own case as hopeless, and particularly adverted to a symptom, which he called the *metallic tinkling* of Laennec; applying that term to a sound connected with his own respiration, and which, he said, was always present to his ear: he compared it to the "faint vibrations of air caused by the gentle stroke of the hammer of a silver bell." He said it was "a death-watch forewarning him of his doom;" and, with much composure, gave Dr. Boott several directions to be attended to after his death. Yet, with an inconsistency, affecting in such circumstances, he spoke with pleasure, two days afterward, of returning to his London practice, and encountering all its anxieties and fatigues. He revived a little, paid a short visit to the north of England, and then, worn and emaciated as he was by long illness, and hovering on the brink of the grave, he actually did resume his practice, although in less than three weeks he again gave it up, never to resume it again. There is something so remarkable and at the same time so painfully interesting in this, the closing scene of his life, that we must extract it from the many details of his long decline which Dr. Boott has with so much feeling related:

— " he returned to London on the 1st of November, (1829,) broken in spirit and fast fading away.

"It was most affecting to witness the persevering but ineffectual efforts he made to rally his sinking energies, and to enter once more upon practice. His mind seemed to infuse vigour into his wasted and enfeebled frame; and for two or three weeks he was much occupied in visiting patients, in and for a distance of several miles from town. But he came home always in a state of great exhaustion; and it was painful to observe his instinctive promptness to attend to the calls of duty, blended with the incapacity of exerting himself without greatly aggravating his sufferings. He could not be persuaded to abandon all thoughts of his profession; and it seemed a relief to him to feel that he was yet capable in some degree of being useful to his family.

"On the 19th he sent an urgent message to me, in the evening. I found him sitting in a corner of his drawing-room, remote from the fire. He told me that he had taken a warm bath, and that he had discovered while in it a fracture of one of his ribs; and he seemed wholly absorbed with the idea that this neglected injury was the origin of his sufferings. The suspicion of this injury, which was entirely groundless, prevailed for a short time; so that he became fearful of making any unnecessary exertion. A few days after this he declined seeing more patients; and, after some feeble attempts to rally himself by exercise in his carriage, and taking short walks on Highgate Hill, he took to bed.

"The last day he rose from it was the 1st of December. I visited him the next morning in his chamber, and he said that he should never leave it. He was in a state of perfect composure of mind, and fully resigned to his fate. On the 3d he told me he might live ten days; that he had done all he could to combat successfully against the disease under which he laboured, but that it had been in vain; and there now remained but one duty more, and that was to rally all life's energies to die."

Those who have ever watched a relative or a friend slow sinking under the oppressions of consumption, whilst yet life was in its prime, have most probably witnessed scenes not unlike those on which Dr. Boott has dwelt with all the earnestness of friendly affection. The mind, calm and detached from all that ruffled it in the world, attains a rapture of repose before death; and, under the slight excitement generally present, the tongue gives utterance to reflections so pure and beautiful, so clear and unworldly, that they seem oracular and sacred. The description of the last days of Dr. Armstrong, whose life was exactly prolonged to the ten days he had spoken of as possible to him, is deeply interesting; and the portraiture of his private and domestic character, which was most simple and engaging, and untinged with any taint of the fashionable affectations that so much disfigure the medical character, is such as no one can read without feeling the vain and common regret that so affectionate a husband, so good a father, so kind a friend, and so benevolent a physician, should so soon have been taken from those who looked up to and relied upon him.

Not many days before his death, the stethoscope was applied to his chest by Dr. Thomas Davies, and the precise accuracy of the diagnosis was verified by an examination after his decease. A large tubercular excavation occupied the upper third of the left lung, capable of containing from twelve to sixteen ounces of fluid; and the remaining portion of the lung was filled with tubercles in all their stages. The upper half of the right lung also was filled with tubercles, accumulated in rounded masses, the interval between the masses being tolerably healthy: its apex contained an excavation capable of holding a small-sized walnut. Disease so extensive had probably been long in progress, and must have affected not his bodily health only, but in some degree that of his mind, so as, in our opinion, to account for some of the peculiarities to which we have, not disrespectfully we hope, pointed.

The pious office of preserving and publishing his Lectures has been performed by Mr. Rix, with singular ability. We respect even the *enthusiasm* of a student for his preceptor; and if we have noticed passages with disapprobation, which might have been left out without detriment to the lectures, we can easily comprehend Mr. Rix's motives for abstaining from the mutilation; and, with these defects, we cheerfully acknowledge that the volume is still extremely valuable.

As respects Dr. Boott's elaborate work, we have limited our remarks to the Memoir of Dr. Armstrong, and to that part of the first volume which relates to Dr. Armstrong's views of the fever of this country. The remainder of the volume is taken up with chapters full of information relating to the yellow fever, to the fevers of the southern, midland, and eastern states of North America, and to the fevers of the city and state of New York. The second volume is devoted to the consideration of the plague, as it has appeared in Egypt, Syria, Holland, France, and London; and of the fevers of Italy, of the Mediterranean, of Paris, and of Great Britain. The section relative to the fevers of Paris comprehends an able analysis of the work of M. Louis; and every section contains facts and observations, to which few medical readers can refer without instruction. The views which they are induced to support form a distinct matter of enquiry from that which has occupied our attention, and which has rather related to the personal character and labours of Dr. Armstrong.

Our opinion of Dr. Armstrong, in all the relations of private life, after reading Dr. Boott's affecting memoir, is such as no language, except that of unfeigned respect and admiration, could express. Our opinion of him as a theoretical and practical physician may be gathered from our previous observations. If we have, in some respects, taken a less flattering view of his qualifications than his biographer has done, it has been with regret that we have found ourselves compelled to dissent from one whose account of his deceased friend, and of that friend's labours, is the evident production of an accomplished physician, whose sensibility, learning, and opportunities, peculiarly fitted him for the task; and whose friendly partialities, if such they be, do him no dishonour. If we did not believe that the example of Dr. Armstrong, and some of the lessons which he taught so eloquently, had produced unfavorable effects on the practitioners and students of his time, had in some degree lowered the standard of attainment which should ever be, and generally is, associated with eminence, had induced an undue disregard even for medical learning, and tended to justify to his imitators a contented reliance on mere personal experience, we should not have felt ourselves called upon to criticise his life and writings.

The impression we entertain is, still, that he was justly eminent as a practitioner, and more than commonly qualified by the habits of his mind, and by his disposition, for exercising the patient and unostentatious duties of his profession; that he was diligent, observing, perspicacious, humane, and to his profession enthusiastically attached. It was partly the misfortune of his education, and partly incidental to his sanguine temperament, that he was ever too easily induced to think himself original, and felt an almost restless eagerness to see everything in a new light. The early traits of his character as an author and practitioner, which were developed in his Publications on Puerperal Fever, seem to us to be discernible throughout his whole life. In all his subsequent compositions we remark the same powers of observation and description; the same felicitous adaptation of practice to the particular disease before him; the same belief that whatever he himself knew at the actual time was the limit at that time of other men's knowledge; the same self-complacent depreciation of his real or supposed opponents; and the same air of being sacrificed to the cause of practical truths, unwelcome to the speculative and the learned. Unlike the generality of students, he looked back, without gratitude, affection, or respect, to the place of his medical education. Of his teachers there, he retained a filial recollection of Dr. Hamilton alone; and it is most probable that, for some reason or other, he saw little other practice in the Infirmary. With the value of the clinical lessons to be gained in it he seems to have been altogether unacquainted. Yet to Edinburgh his education was strictly limited; and it is to be regretted that, with his industrious habits and enquiring mind, his straitened circumstances, when a student, should have deprived him of the advantages then peculiar to that celebrated school of medicine, and which would have enabled him to lay a wider and better foundation for that practical experience which it was his after-delight to build up.

He wrote fully, and somewhat dogmatically, on fever, before his acquaintance with the forms of that disease, which receives modifications from so many circumstance, could guard him from errors which it gave him much subsequent trouble to controvert; and, vacillating evermore between extreme opinions, he never acquired a firm hold of those to which correctness could give stability. He began a contagionist; or, rather, he began by believing human contagion to be the sole cause of genuine typhous fever: he afterward admitted the parallel claims of malaria, or marsh effluvium; but, warming with the discussion, and thus seemingly disqualified for temperate enquiry, he was at length strongly inclined to abandon, if he did not indeed abandon, contagion altogether. His practical foibles were perhaps of deeper consequence. He began as an advocate for large bleedings, and he alternately or successively advocated large doses of calomel, of opium, of colchicum. His unprepared pupils were amazed by the simplicity and uniformity of practice now first unfolded to them. For them no longer was there to be or doubt, or uncertainty, or hesitation, or failure. He, and he alone, without the learning of colleges, had discovered the true key of practice. Great truths, they knew, did ever find slow acceptance: discoverers were generally denied their full merit: such had ever been the reluctant homage paid to the benefactors of mankind; but their merits were acknowledged after their death, and their reputation was enduring. Of the applicability of all this to himself, Dr. Armstrong felt the most undoubting conviction: he affirmed it with a warmth, a solemnity, a pathos, which moved his hearers, and drew tears from their eyes and his own. The consequences were curious: his pupils went forth full of confidence, and full of contempt for learning; they were armed with specific plans of treatment, which, directed by their sagacious and original observation, would never fail. Meantime he, their industrious preceptor, was ever catching new rays of information, and continually modifying those axioms which he and they had deemed immutable, until, towards the close of his active life, there were few practical opinions of his early years which he had not given up: his premature confidence had given way to unreasonable scepticism, and, in the estimation of those who met him in consultation, his practice, from being bold and as it were settled for ever, had become, by successive changes, in the most remarkable degree feeble and inert.

These faults we have dwelt upon, from no unkindness, nor from any unwillingness to wound the unresisting dead, nor to prolong the memory of what detracted from the usefulness of Dr. Armstrong; but from a conviction that they operated unfavorably on the character of students of medicine, and on the London school, to the weakness and prejudices of which they came but too much recommended. The peculiarities of that school had too long been those which Dr. Armstrong encouraged; empirical confidence and theoretical scepticism; a fondness for bold specifics, and an excessive distrust of reasoning, under the denounced name of theory. Not learning alone, but common literary acquirement, was then in some disrepute; practical knowledge was extravagantly and ignorantly extolled; andi n these particulars, certainly, Dr. Armstrong was no reformer.

We speak of days upon which new days have now arisen. The London school is now taking higher ground. But students may learn from the rapid rise and decline of Dr. Armstrong's opinions and authority as a teacher and guide, from his fugitive fame, and almost ineffectual toils, that whatever talents are possessed, and whatever diligence is exercised, medical truths are not to be hastily attained; and that the most acute observations of youth can only acquire permanent value by patient reflection when years have added increase to knowledge and afforded oportunities of wider observation.

---

